# Supplementary material for: Isolation of Two New Phenolic Glycosides from Castanopsis chinensis Hance by Combined Multistep CC and HSCCC Separation and Evaluation of Their Antioxidant Activity
Source: Molecules. 2023 Apr 10;28(8):3331. doi: 10.3390/molecules28083331 (PMC10143513; doi:10.3390/molecules28083331)
Supplement: Supplementary file 1 [file molecules-28-03331-s001.zip › molecules-2278870-supplementary.pdf]

Article

Isolation of Two New Phenolic Glycosides from *Castanopsis chinensis* Hance by Combined Multistep CC and HSCCC Separation and Evaluation of Their Antioxidant Activity

Ya-Feng Wang, Ping Lin, Yong-Lin Huang *, Rui-Jie He, Bing-Yuan Yang and Zhang-Bin Liu

Guangxi Key Laboratory of Plant Functional Phytochemicals and Sustainable Utilization, Guangxi Institute of Botany, Guangxi Zhuang Autonomous Region and Chinese Academy of Sciences, Guilin 541006, China
* Correspondence: hyl@gxib.cn

Abstract: The characteristics of high polarity and susceptibility to oxidation in phenolic glycosides increase the difficulty of their separation from natural products. In the present study, two new phenolic glycosides with similar structures were isolated from *Castanopsis chinensis* Hance using a combination of multistep CC and high-speed countercurrent chromatography. Preliminary separation of the target fractions was carried out by Sephadex LH-20 chromatography (100–0% EtOH in H_2O). High-speed countercurrent chromatography with an optimized solvent system of N-Hexane/Ethyl acetate/Methanol/Water (1:6:3:4, *v*/*v*/*v*/*v*) with a satisfactory stationary phase retention and separation factor was used for further separation and purification of the phenolic glycosides. Consequently, two new phenolic glycoside compounds were obtained with purities of 93.0% and 95.7%. 1D-NMR and 2D-NMR spectroscopy, mass spectrometry, and optical rotation were employed to identify their structures, which were assigned as chinensin D and chinensin E. The antioxidant and α-glucosidase inhibitory activities of these two compounds were evaluated using a DPPH antioxidant assay and a α-glucosidase inhibitory assay. Both compounds showed good antioxidant activity with IC_{50} values of 54.5 ± 0.82 μg/mL and 52.5 ± 0.47 μg/mL. The α-glucosidase inhibitory activity of the compounds was poor. The successful isolation and structure identification of the two new compounds provides materials not only for a systematic isolation method of phenolic glycosides with similar structures, but also for the screening of antioxidants and enzyme inhibitors.

Keywords: phenolic glycosides; *Castanopsis chinensis* Hance; high-speed countercurrent chromatography; antioxidant activity

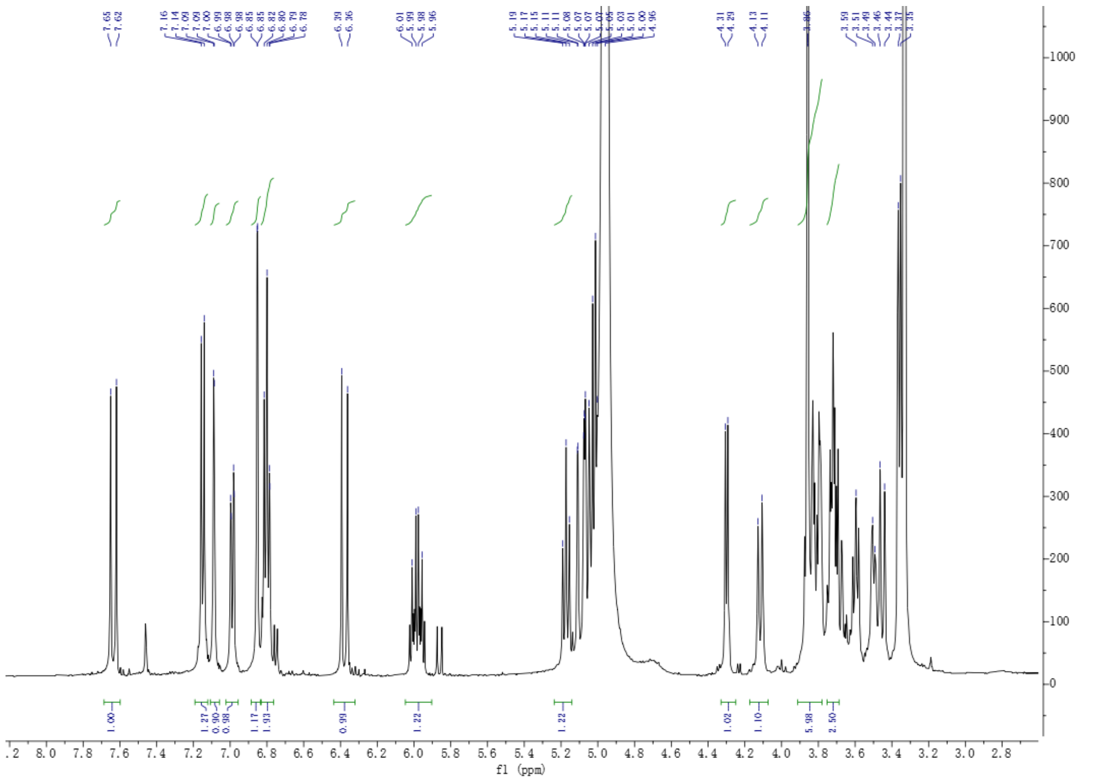

Figure S1 The ^{1}H NMR spectrum of compound **1**

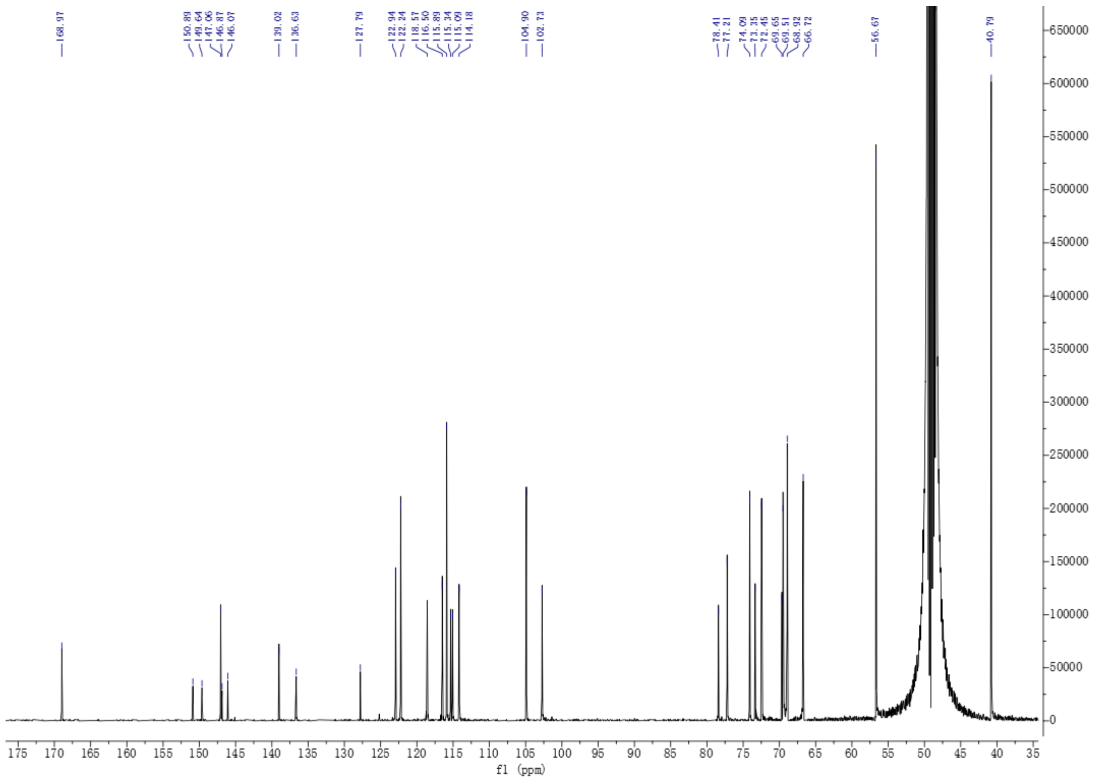

Figure S2 The ^{13}C NMR spectrum of compound **1**

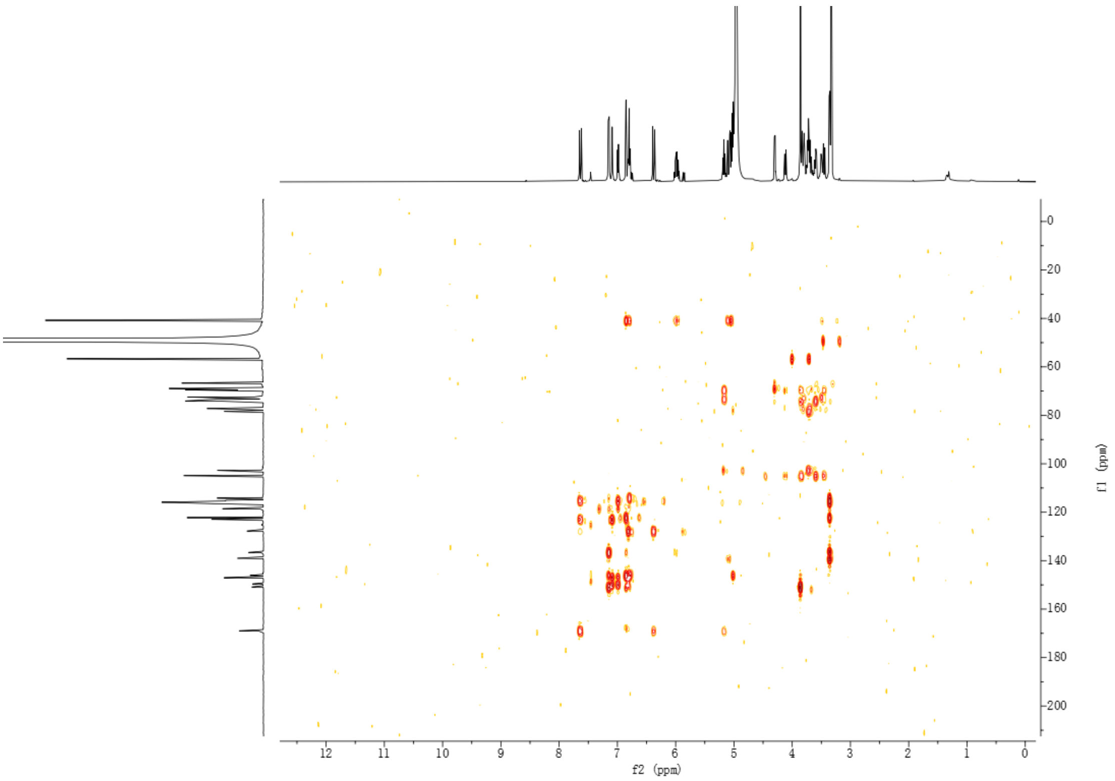

Figure S3 The HMBC spectrum of compound **1**

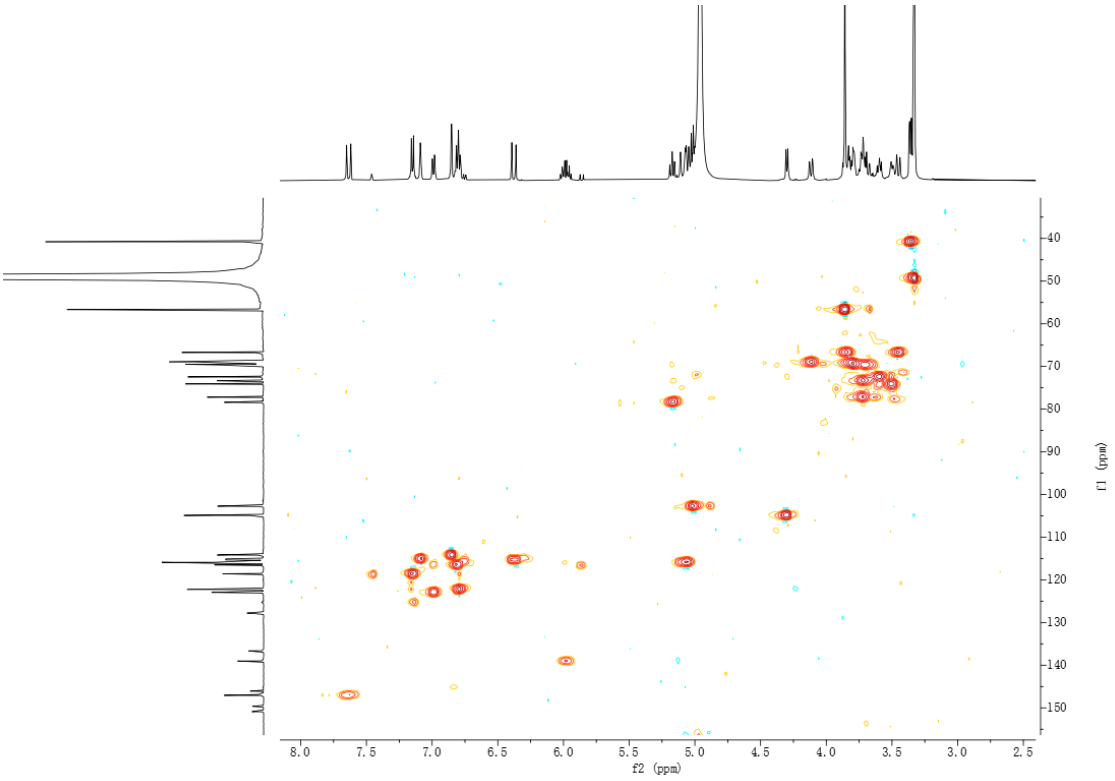

Figure S4 The HSQC spectrum of compound **1**

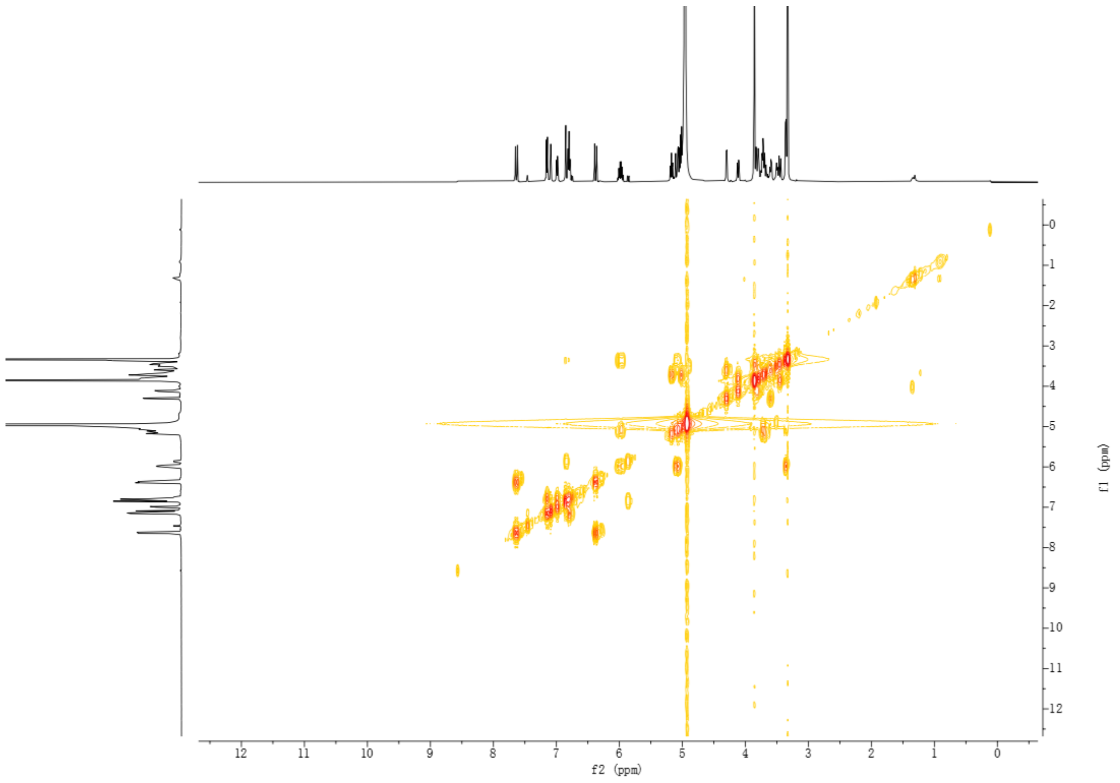

Figure S5 The H-H COSY spectrum of compound **1**

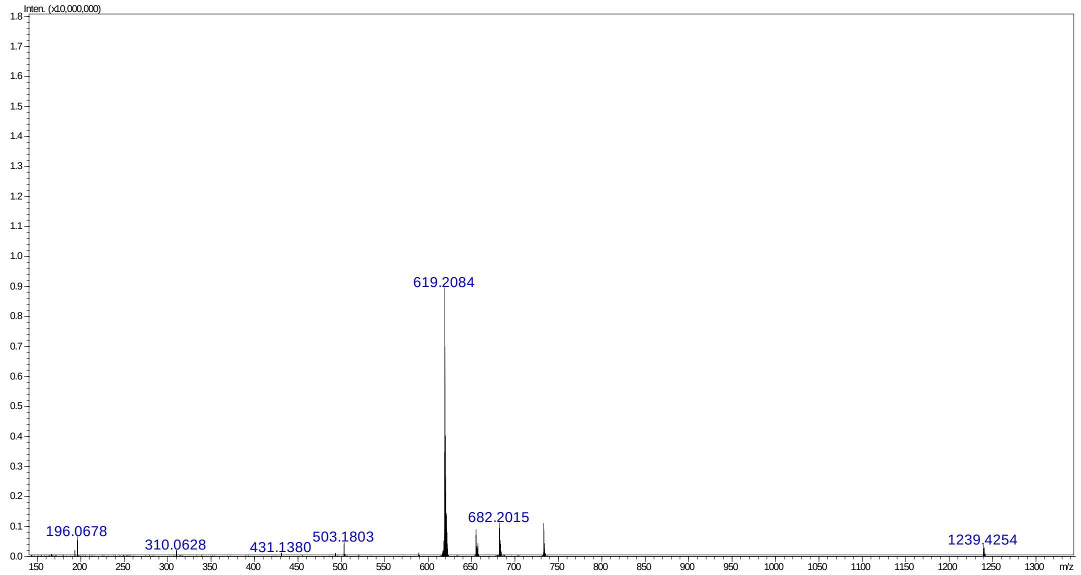

Figure S6. The LC-MS/IT-TOF spectrum of compound **1**

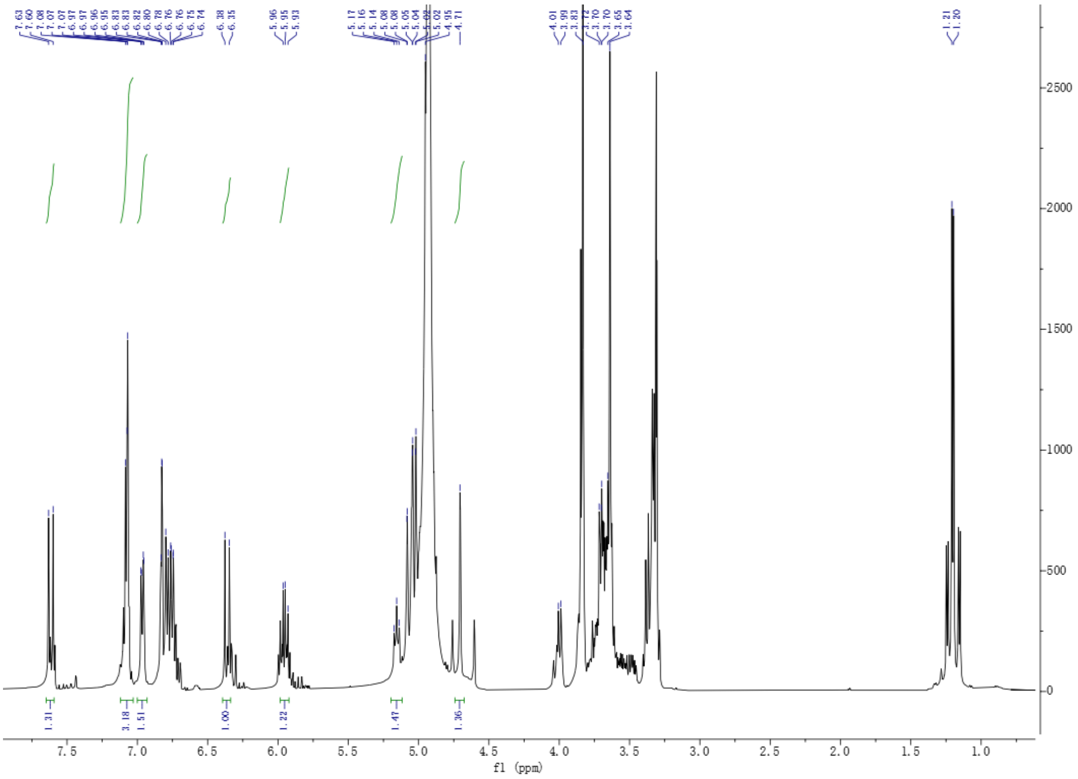

Figure S7 The ^{1}H NMR spectrum of compound **2**

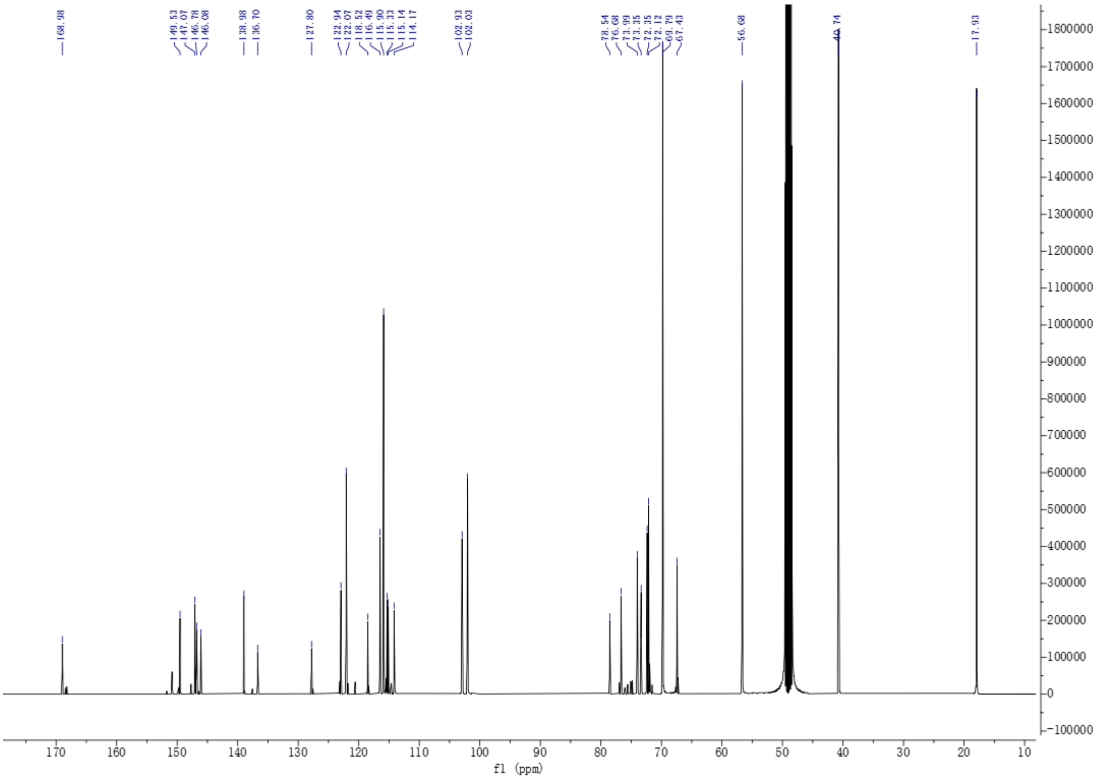

Figure S8 The ^{13}C NMR spectrum of compound **2**

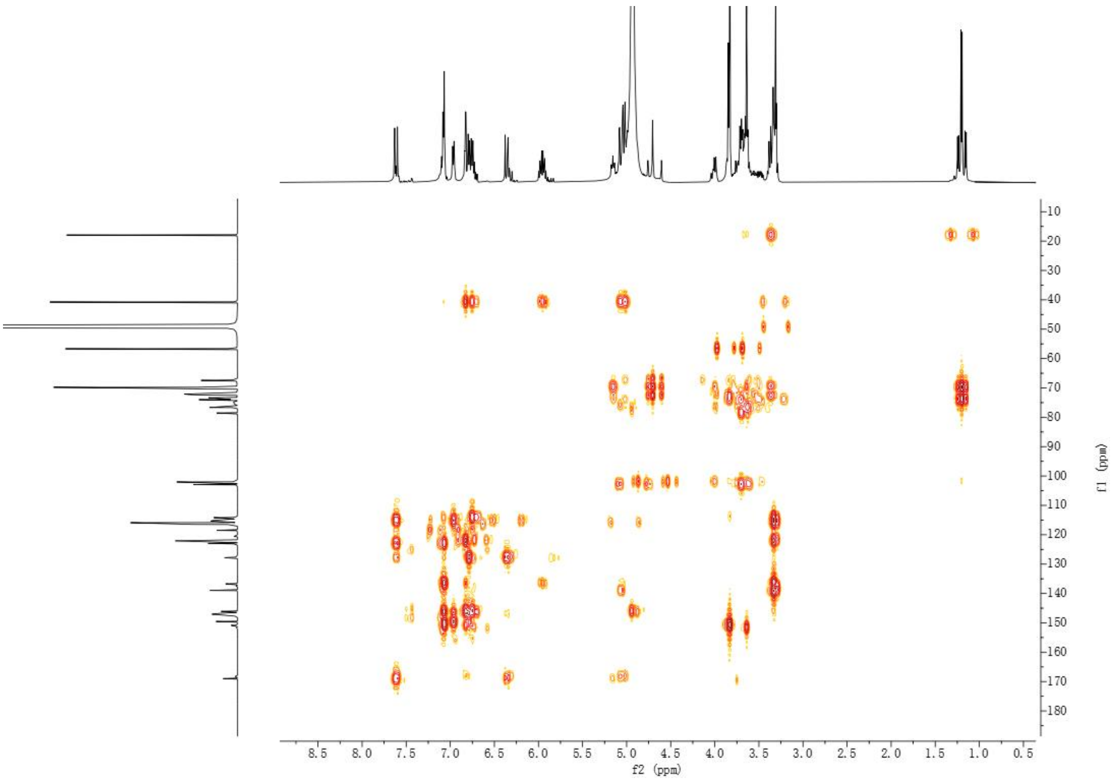

Figure S9 The HMBC spectrum of compound **2**

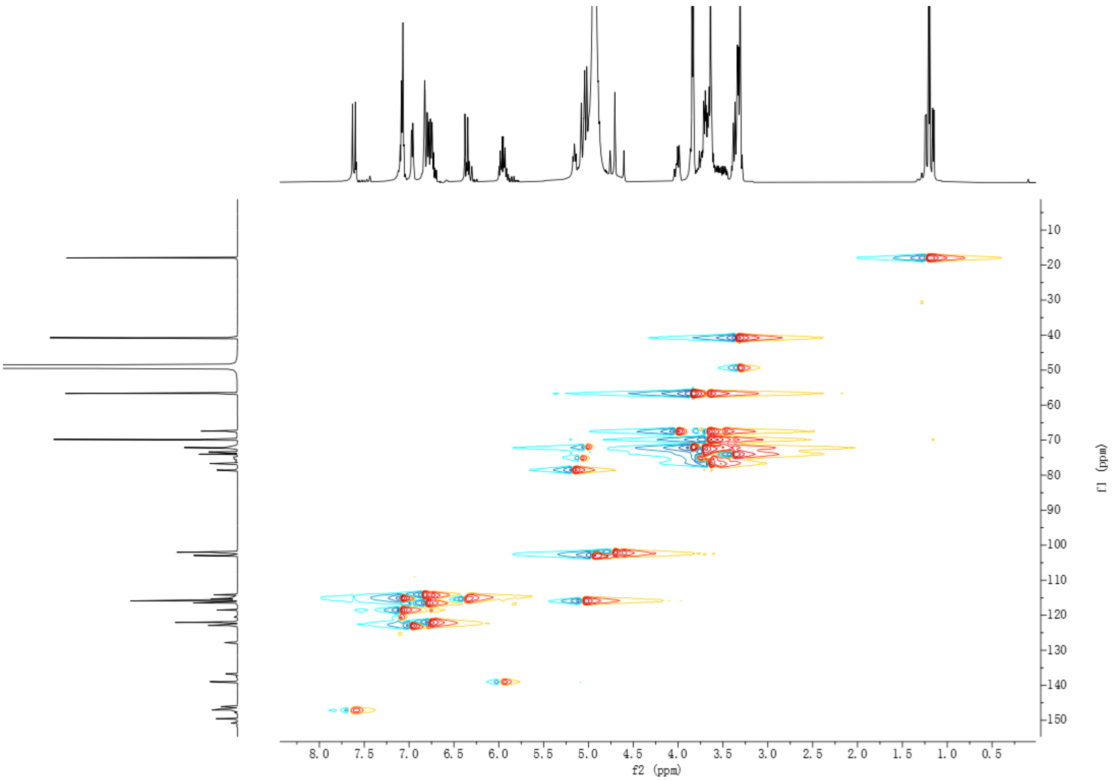

Figure S10 The HSQC spectrum of compound **2**

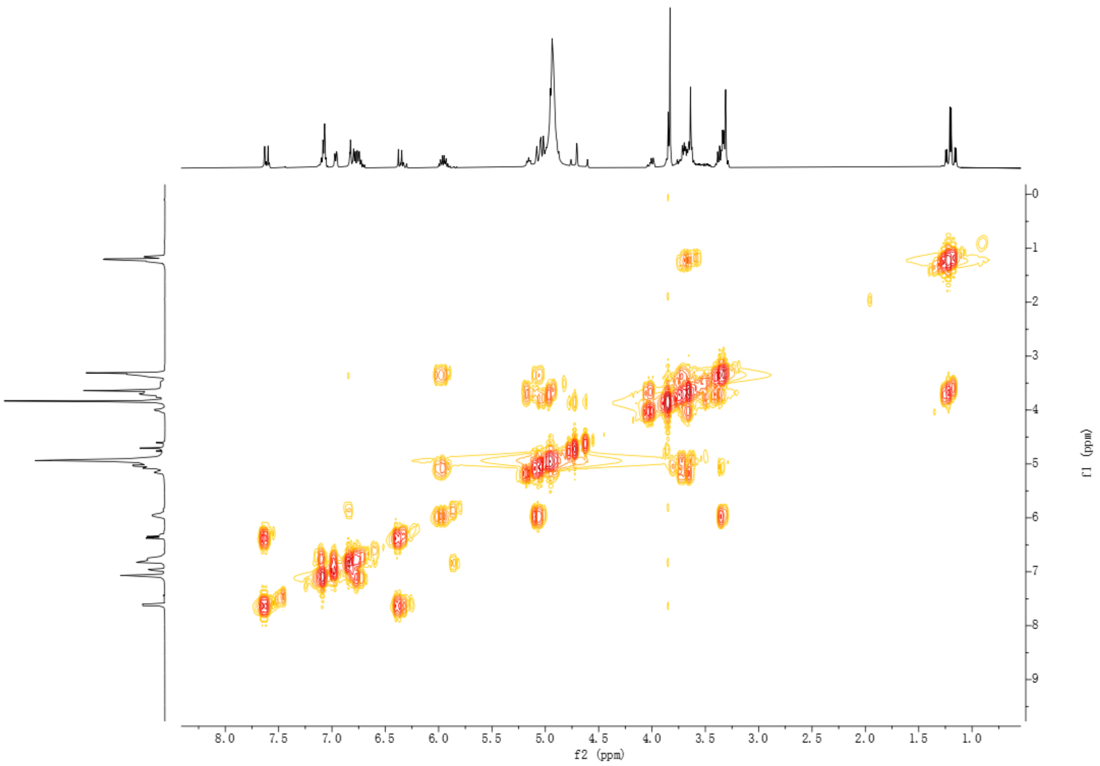

Figure S11 The H-H COSY spectrum of compound **2**

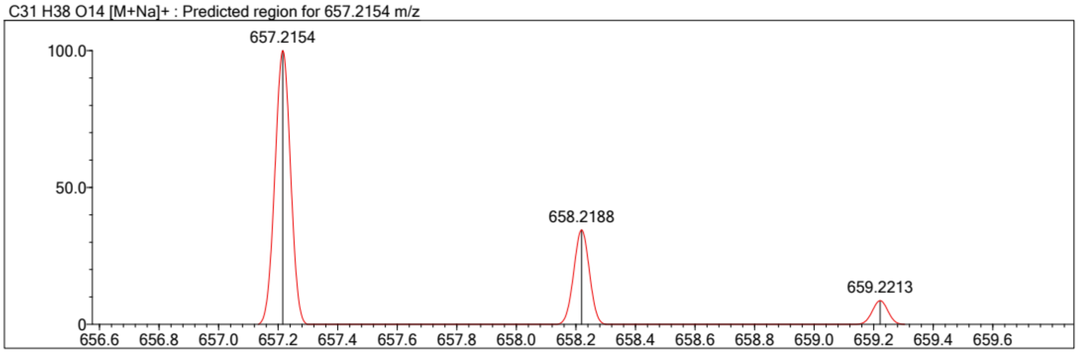

Figure S12 The LC-MS/IT-TOF spectrum of compound **2**
